# Effects of acute ingestion of caffeinated chewing gum on performance in elite judo athletes

**DOI:** 10.1186/s12970-021-00448-y

**Published:** 2021-06-19

**Authors:** Aleksandra Filip-Stachnik, Robert Krawczyk, Michal Krzysztofik, Agata Rzeszutko-Belzowska, Marcin Dornowski, Adam Zajac, Juan Del Coso, Michal Wilk

**Affiliations:** 1 Institute of Sport Sciences, The Jerzy Kukuczka Academy of Physical Education in Katowice, Katowice, Poland; 2 College of Medical Sciences, Institute of Physical Culture Studies, University of Rzeszów, Rzeszów, Poland; 3 Faculty of Physical Education, Gdańsk University of Physical Education and Sport, Gdańsk, Poland; 4 Centre for Sport Studies, Rey Juan Carlos University, Fuenlabrada, Spain

**Keywords:** Ergogenic aid, Combat sport, Elite athlete, Exercise performance, Stimulant

## Abstract

**Purpose:**

Previous investigations have found positive effects of acute ingestion of capsules containing 4-to-9 mg of caffeine per kg of body mass on several aspects of judo performance. However, no previous investigation has tested the effectiveness of caffeinated chewing gum as the form of caffeine administration for judoists. The main goal of this study was to assess the effect of acute ingestion of a caffeinated chewing gum on the results of the special judo fitness test (SJFT).

**Methods:**

Nine male elite judo athletes of the Polish national team (23.7 ± 4.4 years, body mass: 73.5 ± 7.4 kg) participated in a randomized, crossover, placebo-controlled and double-blind experiment. Participants were moderate caffeine consumers (3.1 mg/kg/day). Each athlete performed three identical experimental sessions after: (a) ingestion of two non-caffeinated chewing gums (P + P); (b) a caffeinated chewing gum and a placebo chewing gum (C + P; ~2.7 mg/kg); (c) two caffeinated chewing gums (C + C; ~5.4 mg/kg). Each gum was ingested 15 min before performing two Special Judo Fitness Test (SJFT) which were separated by 4 min of combat activity.

**Results:**

The total number of throws was not different between P + P, C + P, and C + C (59.66 ± 4.15, 62.22 ± 4.32, 60.22 ± 4.08 throws, respectively; p = 0.41). A two-way repeated measures ANOVA indicated no significant substance × time interaction effect as well as no main effect of caffeine for SJFT performance, SJFT index, blood lactate concentration, heart rate or rating of perceived exertion.

**Conclusions:**

The results of the current study indicate that the use of caffeinated chewing gum in a dose up to 5.4 mg/kg of caffeine did not increase performance during repeated SJFTs.

## Introduction

Caffeine is recognized as the most commonly used psychoactive substance in the world [1] and is widely utilized by elite athletes as an ergogenic aid to increase physical performance during training and competition [2]. Indeed, recent scientific reviews and meta-analyses confirm the benefits of this substance on various types of exercise including aerobic-based [3], anaerobic-based [4], and strength/power exercise activities [5]. Moreover, the ergogenic effects of caffeine have been observed in intermittent sport disciplines, such as team sports [6] and combat sports [7], which require a substantial contribution from both oxidative and non-oxidative metabolism in addition to sport-specific technical and tactical skills.

In most previous investigations confirming the ergogenic effects of caffeine in sports performance, this stimulant was provided in doses from 3 to 9 mg per kg of body mass (i.e., mg/kg) in the form of anhydrous caffeine administered in a gelatin capsules. However, in the sport setting, caffeine is generally consumed in the form of caffeinated beverages such as coffee or tea, pre-work out supplements or in capsules/pills, although there are several other sources of caffeine [8]. Interestingly, an alternative method of caffeine delivery via chewing gum may provide an advantage over traditional forms of caffeine administration. Caffeine via chewing gum offers a different pharmacokinetic profile over the ingestion of caffeine in capsules, which results in an earlier increase in blood plasma caffeine concentration, usually between 5 and 15 min from intake [9]. Moreover, chewing gum allows caffeine to be absorbed directly into the bloodstream through the buccal mucosa, thereby bypassing hepatic metabolism [9]. This form of caffeine absorption may minimize the risk of gastrointestinal disorders in athletes. Regarding this issue, the use of caffeinated chewing gum in doses between 2 and 6 mg/kg has been found effective in increasing performance in several types of exercise, such as cycling [10–12], team sports-specific tests [13, 14], endurance running [15, 16] and jumping performance [17] although this is not always the case [18, 19].

Despite the evidence of ergogenic effects of caffeinated chewing gum, there is no study testing the efficacy of this form of caffeine administration in combat sports such as judo. The use of caffeinated chewing gum in judo may be more beneficial than the use of caffeine capsules because judo tournaments consist of elimination rounds habitually performed without a fixed schedule. Additionally, there is a need of performing several judo combats within the same competition day. To date, the studies examining the ergogenic effects of caffeine in judo used caffeine capsules [7, 20–23] or caffeine dissolved in water [24]. In these investigations, the acute intake of caffeine in a dose of 4-to-9 mg/kg was effective to enhance several aspects of judo performance during simulated combats [20, 23, 24], although the effect of this supplementation protocol of caffeine administration seems ineffective after rapid weight loss [7] and in women [22]. However, the use of a single administration of caffeine may have reduced applicability to the context of a real judo competition, where several combats take place in one day. In this context, repeated dosing of caffeine before each combat may be necessary [25]. Interestingly, Negaresh et al. [26] showed that a repeated-dosing of caffeine (i.e., before each match) improved wrestling performance in the last stages of a 5-match wrestling tournament in comparison to a single administration of a particular dose of caffeine before the tournament. This suggests that ingestion of caffeine in smaller doses prior to each combat during a tournament may offer greater performance enhancement than the use of a single and larger dose before competition.

Therefore, the aim of this study was to examine the effects of the ingestion of caffeinated chewing gum on judo performance in elite athletes. To improve the applicability of the experiment, we tested two caffeine supplementation protocols with a single and repeated dosing of caffeine that provided two different doses of caffeine. It was hypothesized that caffeine supplementation protocols would increase judo performance in comparison to the administration of a decaffeinated/placebo chewing gum.

## Materials and methods

### Study participants

Power analysis indicated that a minimum sample size of 9 participants should be included in the study in order to detect an effect size (ES) of 0.5, obtained from a study examining acute effects of caffeine on judo performance in Special Judo Fitness Test SJFT [21]. Power was analyzed using the following variables: the analysis was set to repeated measures ANOVA, within factors, the required power was set to 0.80, alpha was set to 0.05, and the correlation between repeated measures was set to r = 0.5. This calculation was performed with the G*Power software, v.3.1.9.2 [27]. Therefore, we recruited nine male healthy experienced judoists to participate in the study. The following anthropometric measurements were taken: height (WPT-60/150OW, Radwag, Poland), body mass and body fat percentage (InBody 370, Poland). Main characteristics of the study sample are depicted in Table 1. Participants were recruited from the Academic Sports Club of AZS AWF Katowice and testing was conducted during the competitive season. All athletes selected for the research were black belts, competed at the national and international level and were members of the Polish national team. The inclusion criteria were as follows: (a) free from neuromuscular and musculoskeletal disorders; (b) black belt and at least “good” level in the SJFT [28] (c) no medication nor dietary supplements use within the previous month; (d) self-described satisfactory health status. Participants were excluded if they reported (a) positive smoking status; (b) potential allergy to caffeine. All participants had previous experience in performing the SJFT test during training and/or investigations. None of the participants had previously used caffeinated chewing gum. The study protocol was approved by the Bioethics Committee for Scientific Research, at the Academy of Physical Education in Katowice, Poland, (3/2019) according to the ethical standards laid down in the 1964 Declaration of Helsinki and its later amendments. All participants provided their written informed consent prior to participation in this study.

**Table 1:** Main participants’ characteristics

Age [years]	23.7 ± 4.4
Body mass [kg]	73.5 ± 7.4
Height [cm]	174.3 ± 4.0
Body Fat [%]	11.1 ± 4.0
Judo training experience [years]	15.6 ± 4.0
Habitual caffeine intake [mg/kg/day]	3.1 ± 1.3

### Pre experimental standardization

Prior to the first experimental trial, participants were instructed to maintain their usual hydration and dietary habits (including pre-workout meal) and habitual caffeine intake during the study period. In addition, the participants registered their food intake using “MyFitnessPal” software [29] 24 h before the first experimental trial. To produce a within-subject standardization of diet, participants replicated the same dietary pattern before the second and third trials. Habitual caffeine intake was measured by using a modified version of the validated questionnaire by Bühler et al. [30] that recorded the type and amount of caffeine-containing foods and dietary supplements. Habitual caffeine intake was assessed for the four weeks before the start of the experiment, following previous recommendations [31]. Participants were also asked to refrain from any source of caffeine and alcohol 24 h before each experimental trial and not to perform strenuous exercise in the 24 h before testing.

### Experimental design

To investigate the effect of caffeine consumption via caffeinated chewing gum on judo performance the participants underwent a randomized, double-blind, placebo-controlled crossover experiment where each participant acted as his own control. Each participant took part in three identical experimental trials that included the ingestion of two chewing gums and several judo-specific performance measurements. The trials differed in the type of gum ingested as follows: (a) ingestion of two non-caffeinated (i.e., placebo) chewing gums (P + P; 0 mg/kg of caffeine); b) a caffeinated chewing gum and a placebo chewing gum (C + P; 200 mg of caffeine or ~ 2.7 mg/kg of caffeine); c) two caffeinated chewing gums (C + C; 400 mg of caffeine or ~ 5.4 mg/kg of caffeine). The trials were separated by seven days to allow complete recovery and substance wash-out.

**Fig. 1:**
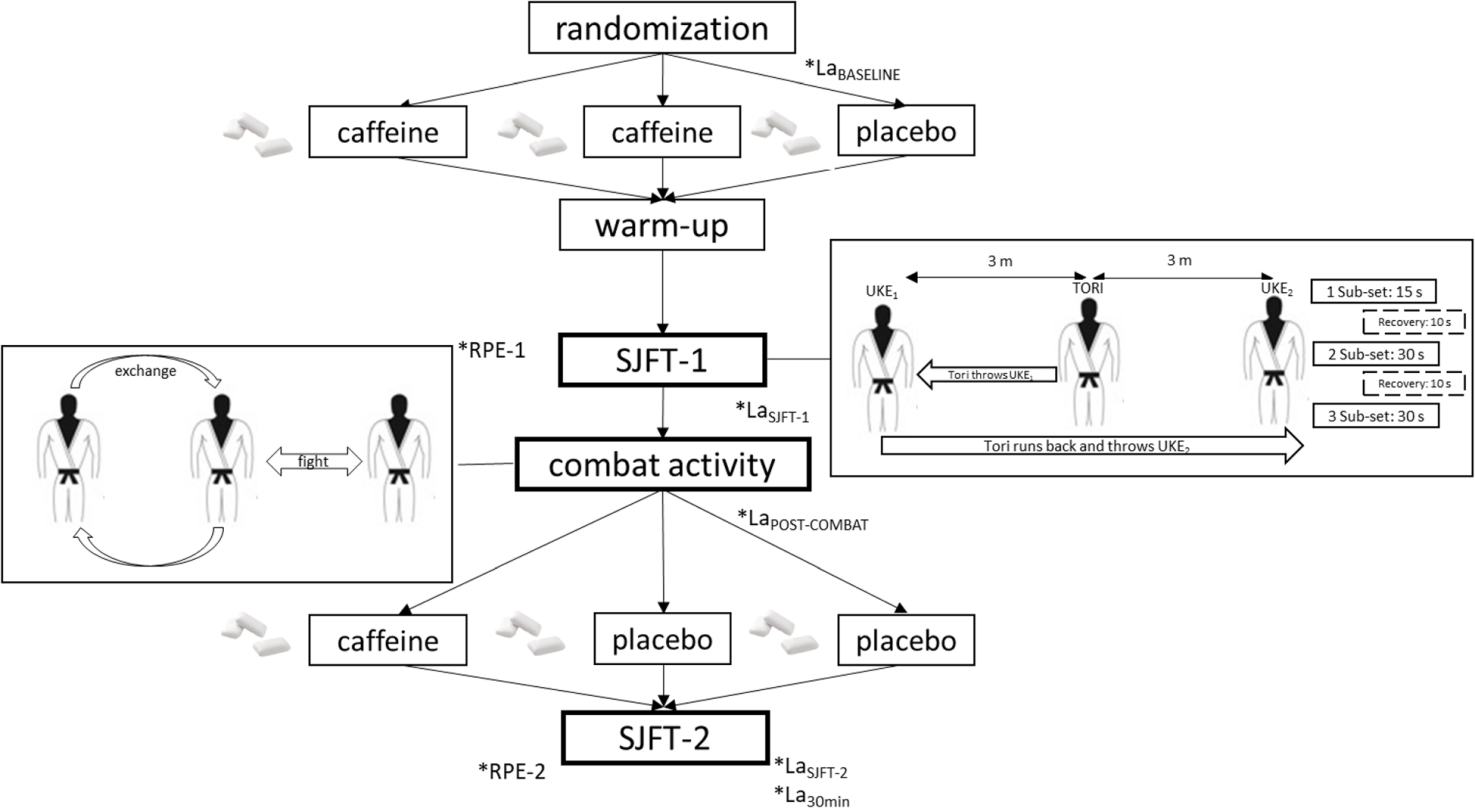
Study design. SJFT-1, SJFT-2; Repetitions #1 and #2 of the Special Judo Fitness test; RPE-1, RPE-2; Measurements of the rating of perceived exertion obtained just after finishing the first and second SJFT, respectively; La_BASELINE_measurement of blood lactate concentration measured before warm up for baseline ; La-_SJFT-1_– blood lactate concentration 3 min after finishing the first SJFT; La_POST-COMBAT_blood lactate concentration after combat activity; La-_SJFT-2_– blood lactate concentration 3 min after finishing the second SJFT; La_30min_– blood lactate concentration 30 min after the end of the second SJFT

Upon arrival to the laboratory, a blood sample was obtained to assess blood lactate concentration at baseline (BIOSEN C-line; EKF, United Kingdom). Then, participants wore their judogis and ingested the first chewing gum. Afterwards, the participants performed a 15-min standardized warm up, simulating a pre-competition warm up. Then, the participants performed the first SJFT, as described below. After the first SJFT, blood lactate concentration and the rating of perceived exertion (RPE; using the 6–20-point Borg scale; [32]) were obtained, while heart rate (Wearlink, Polar, Finland) was evaluated at the end of the SJFT and 1 min into recovery. After 5 min of passive recovery, participants performed a 4-min simulated combat activity with no performance measurements and blood lactate concentration was measured again. Immediately after the combat, the second chewing gum was consumed and then participants rested for 15 min before performing the second SJFT. The rating of perceived exertion was measured immediately after the second SJFT, heart rate was measured immediately after the second SJFT and 1 min into recovery, while blood lactate concentration was measured after the second SFJT and 30 post exercise. All testing was performed at the Strength and Power Laboratory of the Academy of Physical Education in Katowice, Poland under controlled ambient conditions. All experiments took place at the same time of the day (18:00–20:00 in the afternoon) during their habitual judo training time. Figure 1 contains a description of the experimental design used for the experiment.

### Administration of caffeinated and placebo chewing gum

In each trial, participants chewed the gums for 5 min and were then required to expectorate the chewed gum into a container. The gums were ingested 15 min before the onset of each SJFT considering that a large increase in plasma caffeine concentration after administration of 200 mg of caffeine via chewing gum occurs between 5 and 15 min after administration [8, 9]. During each intake, the gum contained 200 mg of caffeine from a commercially available chewing gum (Military Energy Gum; MarketRight Inc., Plano, IL, USA). The placebo was a commercially available non-caffeinated chewing gum, similar in taste, shape, and size. The chewing gums were placed in an opaque container in order to blind participants and experimenters from the conditions under investigation. Verbal questioning after every SJFT indicated that participants were unable to distinguish between the caffeine and non-caffeine-containing chewing gum (odds no greater than chance or 50:50).

### Special judo fitness test and combat activity

The SJFT is considered a reliable and reproducible mean to measure judo performance and it was performed according to previous guidelines [28]. The SJFTs consisted of 3 consecutive rounds (first round with 15 s of duration, followed by two rounds of 30 s of duration) with 10-s rest intervals between them. In each round, the judoist under investigation (*Tori*) performed the highest number of *Ippon-seoinage* throws on two partners (*Uke*) which were of similar body mass and they were always the same for a particular *Tori* across the study. To standardize the test among conditions, *Toris* were positioned 6 m apart and the *Uke* was required to run from one *Tori#1* to *Tori*#2 as fast as possible, to throw him using the *Ippon-seoinage* technique, continuing then the run from *Tori#2* to *Tori*#1 to repeat the sequence of running plus throw as many times as possible during the time for each round (Fig. 1). The SJFT performance was measured by the number of complete and valid throws performed in the three rounds, which were confirmed in real time by two independent and experienced coaches blinded to the treatments. The sum of total throws completed in two SJFTs performed in the investigation was also calculated as a measurement of performance. Additionally, the SJFT index was calculated for each SJFT test as follows [28]:

$$Index=\frac{final \ heart \ rate \left(bpm\right)+heart \ rate \ 1 \ {min} \ after \ SJFT \left(bpm\right)}{number\ of \ throws}$$

In each SJFT, heart rate was measured by using a heart rate monitor (Polar, Finland). Five minutes after the first SJFT, the participants performed a 4 min combat activity, where the opponent changed every 2 min of the fight in order to induce fatigue of the central fighter. During combat activity the participants competed with opponents in the same weight category and with a similar sports level relative to their ranking. The participants were compelled to fight with the aim of scoring the most points or winning by ippon, yet the fight was continued regardless of the score. During the SJFTs and combat activity athletes were motivated by members of the research team (two black belt coaches) to exert maximal effort.

### Statistical analysis

All calculations were performed using SPSS (version 25.0; SPSS, Inc., Chicago, IL, USA), and were expressed as means with standard deviations (± SD). Statistical significance was set at p < 0.05. Verification of differences in calorie intake, protein, fat, and carbohydrate ingestion between the P + P vs. C + P and C + C groups was performed using one-way analysis of variance (ANOVA) for repeated measures. Data obtained at baseline or after the combats were also tested with one-way ANOVAs of repeated measures. A two-way ANOVA with repeated measures (condition × time) were used to evaluate the effects of caffeine administration on all measured variables twice during the SFJT tests. Differences in the sum of total throws during the two SJFTs were determined by one-way ANOVA for repeated measures. In the event of a significant main effect, post-hoc comparisons were conducted using the Bonferroni test. Mauchly’s test of sphericity was conducted to test for homogeneity of data and if violated (p < 0.05), the Greenhouse-Geisser adjustment value was used. To test for possible differences between the two doses of caffeine employed in this investigation, values in the total number of throws, the SJFT index, RPE, blood lactate concentration and heart rate were compared by using the related-samples Friedman’s two-way analysis of variance by ranks. Effect sizes (Cohen’s *d*) were reported where appropriate and were defined as large *d* > 0.80; moderate between 0.79 and 0.50; small between 0.49 and 0.20; and trivial as < 0.20 [33].

## Results

The one-way ANOVA indicated no significant differences in energy intake (3286 ± 254, 3288 ± 241, 3298 ± 250 kcal/day; p = 0.17) and in the proportions of protein/carbohydrate/fat (20/52/28, 20/52/28, 20/53/27 %; p = 0.74 for protein; p = 0.77 for carbohydrates; p = 0.88 for fat) in the diet of the judoists between P + P, C + P, and C + C conditions. Table 2 depicts performance and physiological variables during the two repetitions of the SJFT. The two-way ANOVA revealed no main effects of substance and time, nor interaction between these two variables in the number of throws performed in each repetition of the SJFT. As a result, the total number of throws was not different between P + P, C + P, and C + C (59.66 ± 4.15, 62.22 ± 4.32, 60.22 ± 4.08 throws, respectively; p = 0.063). Additionally, there were no main effect nor interaction differences in the SJFT index and in RPE for each repetition of the SJFT (Table 2).

**Table 2:** Performance and physiological variables with ingestion of two non-caffeinated chewing gum (P + P), a caffeinated chewing gum and a placebo chewing gum (C + P; 2.7 mg/kg of caffeine) and two caffeinated chewing gums (C + C; 5.4 mg/kg of caffeine) before executing two repetitions of the Special Judo Fitness Test (SJFT)

Variables		P + P	C + P	C + C	Substance	Time	Interaction
Throws [n]	SJFT-1	29.44 ± 1.94	31 ± 2	30 ± 2.24	0.063	0.111	0.447
	SJFT-2	30.22 ± 2.33	31.22 ± 2.44	30.22 ± 1.99			
SJFT index [%]	SJFT-1	11.55 ± 0.83	11.01 ± 1.1	11.77 ± 2.14	0.099	0.193	0.961
	SJFT-2	11.30 ± 0.92	10.82 ± 0.95	11.50 ± 1.35			
Rate of perceived exertion [arbitrary units]	SJFT-1	17.89 ± 1.36	17.44 ± 2.46	17.22 ± 1.3	0.538	0.153	0.896
	SJFT-2	17.44 ± 1.81	16.67 ± 1.87	16.56 ± 2.19			
Blood lactate concentration [mmol/L]	SJFT-1	13.53 ± 2.46	15.1 ± 1.97	14.03 ± 2.85	0.098	0.869	0.223
	SJFT-2	13.29 ± 2.44	14.66 ± 2.3	14.93 ± 3.6			
Heart rate [bpm]	SJFT-1	192 ± 14	193 ± 17	191 ± 18	0.971	0.525	0.782
	SJFT-2	189 ± 12	190 ± 15	191 ± 13			

All data are presented as mean ± standard deviation for nine elite judoists

Blood lactate concentration at baseline was similar in all conditions (1.57 ± 0.47, 1.72 ± 0.50, 1.62 ± 0.70 mmol/L, respectively p = 0.835). There was no main effect nor interaction in post-SJFT blood lactate concentration (Table 2). Blood lactate concentration after the combat performed between the SJFTs (11.35 ± 3.62, 12.27 ± 2.43, 12.32 ± 3.42 mmol/L, respectively p = 0.733) nor 30 min after the end of the testing were similar among conditions (6.07 ± 3.23, 5.71 ± 2.86, 6.93 ± 2.39 mmol/L, respectively p = 0.078) for P + P, C + P, and C + C.

Heart rate at baseline was similar among conditions (89 ± 11, 84 ± 6, 85 ± 9 bpm, respectively p = 0.308). There was no main effect nor interaction in post-SJFT heart values (Table 2). Additionally, heart rate 1 min after the SJFTs (SJFT-1: 151 ± 15, 147 ± 12, 156 ± 24 bpm and SJFT-2: 151 ± 7, 145 ± 8, 155 ± 20) and after the combat performed between the SJFTs (186 ± 9, 186 ± 12, 186 ± 14 bpm) was not different among conditions (all p > 0.05).

The Friedman’s test showed no significant differences for the total number of throws (p = 0.305), SJFT index (p = 0.489), RPE (p = 0.570), blood lactate concentration (p = 0.416) and heart rate (p = 0.964) between the two doses of caffeine under investigation.

## Discussion

The purpose of this study was to investigate the effects of the ingestion of caffeinated chewing gum on judo performance during two specific judo tests (SJFT) in elite judo athletes. The results of the presented study indicate that the ingestion of caffeinated chewing gum providing two doses of caffeine (2.7 and 5.4 mg/kg of body mass) by using two different protocols (C + P; C + C) did not increase the number of throws performed during the SJFTs when compared to the administration of decaffeinated chewing gum (P + P). In addition, none of the caffeine administration protocols via chewing gum changed the SFJT index, the rate of perceived exertion, blood lactate concentration or post-exercise heart rate. Collectively, the results of the current study suggest that the use of caffeinated chewing gum in a dose up to 5.4 mg/kg did not increase performance during repeated SJFTs.

Several previous studies analyzed the effectiveness of acute caffeine intake in combat sports [34], but only a few focused on judo performance [7, 20–23]. Overall, these investigations showed that the ingestion of a single caffeine capsule or caffeine dissolved in water increased the number of throws in the SJFT [20, 24], or there was an effect of small magnitude that did not reach statistical significance [21]. Additionally, it has been found that this protocol of caffeine administration induced a reduction in the rate of perceived exertion [20] and increased the number of attacks [24] and blood lactate concentration after simulated judo matches [23], and after the SJFT [21] which suggests a higher intensity and higher utilization of anaerobic-based pathways during exercise. In the current experiment, none of these benefits were found after the administration of caffeine via chewing gum, which is contradictory to previous results. The reasons for the differences between investigations can be associated to the administered dose and the habituation to caffeine in the participants of the experiment, in addition to the caffeine supplementation form used. In studies confirming ergogenic effects of caffeine, doses between 4 and 9 mg/kg of caffeine were administered [20, 23, 24]. These doses are above those provided in the C + P protocol (i.e., 2.7 mg/kg), but this does not explain the lack of ergogenic effects of the C + C protocol because the dose administered was 5.4 mg/kg of caffeine. Interestingly, Durkalec-Michalski et al. [24] investigated the effect of three different doses of caffeine (3, 6 and 9 mg/kg) and tested their effects in judoist with different habitual caffeine consumption (consumers and non-consumers). Among those who habitually consumed caffeine, only the dose of 9 mg/kg increased the number of throws during the SJFT while 6 and 9 mg/kg were ergogenic in those unhabituated to the use of caffeine. In the current investigation, all participants were caffeine consumers (Table 1) which may have reduced the efficacy of the C + C protocol, despite that the total dose of caffeine administered was above their habitual intake. Considering all the data collected, it seems reasonable to conclude that the administration of caffeine in caffeinated chewing gum in a dose up to 5.4 mg/kg was ineffective in enhancing SJFT performance in elite judoist habituated to caffeine. Future investigations should determine if higher doses can produce ergogenic effects in this type of elite athletes or whether the ergogenic effects of caffeine are present when investigating elite judoist with low caffeine consumption [21, 24].

The protocol of caffeine consumption may be an important factor affecting the level of acute responses in the presented research. Negaresh et al. [26] compared the effects of caffeine on wrestling performance following different protocols of consumption: a placebo, a high-dose of caffeine (10 mg/kg), a moderate-dose of caffeine (4 mg/kg), repeated-dose of caffeine (2 mg/kg before each fight for a total of 10 mg/kg) or a selective caffeine administration based on performance decrement previously measured (~ 2 mg/kg before each fight for a total of 6.16 ± 1.58 mg/kg). Interestingly, the two protocols that used repeated caffeine doses before each fight yielded the highest improvements in performance, particularly in the last stages of the simulated tournament. This means that, in combat sports, the use of repeated small doses of caffeine before each combat may be the recommended strategy to provide caffeine, instead of a single dose before the first combat. With this repeated dosing protocol, it is probable that athletes do not benefit from caffeine intake during the first combats (as it happened in the current investigation that entailed two SJFT interspersed by a simulated combat), but it will render benefits during the last combats of the competition. To determine if the use of chewing gum offers some benefits over the use of caffeine pills for this protocol of repeated and small dosing requires further investigation.

The ergogenic effects of caffeine can also be related with the sports level of athletes [35]. In our study the participants included elite judoists, based on judo belts and the results of the SJFT, and no previous study considering the effects of caffeine in judo has been conducted on such elite athletes. For highly trained individuals there is less ‘potential for improvement’ after caffeine ingestion because they have reached the upper limits of exercise performance and physical conditioning [36, 37], which may also explain the lack of ergogenic effects following acute caffeine ingestion in this study. The confirmation that caffeine’s ergogenic properties could vary according to training status in judo athletes, may result from the comparison of our results and those obtained in previous research which found a positive effect of caffeine on judo performance [20, 24]. All of the elite judo athletes, who participated in the current investigation reached “good” or “excellent” results in the total throws performed in each SJFT (≥ 27 and ≥ 28 for junior and senior, respectively [28]). In contrast, the number of throws performed in the control/ baseline condition in the study of Astley et al. [20] (23.9 ± 1.7 throws), and Durkalec-Michalski et al. [24] (24.5 ± 2.5 throws) was significantly lower. Moreover, athletes who participated in our study had significantly greater judo training experience than in those two studies [20, 24] (15.6 ± 4.0 vs. 11.00 ± 4.5 years and junior athletes in age 16.1). Similarly, in the study of Lopes-Silva et al. [7] and Felippe et al. [21], where athletes had longer training experience (14.4 ± 8.9 and 15 ± 5 years, respectively), judo performance did not improve after caffeine ingestion. Moreover, only studies performed on younger athletes ( 16.1 ± 1.4  and 21.7 ± 3.7 years, respectively ) [20, 24] showed a positive effect of caffeine, which is contrary to the results of the present study by using judoists of 23.7 ± 4.4 years, as well as to results from previous studies conducted on more experienced participants (25.3 ± 5.7 and 23 ± 5 years, respectively) [7, 21]. Taking into account that peak performance for judo athletes typically occurs when they are 25.4 ± 3.8 years of age (based on World Championships and Olympic Games [38]) it may be suggested that experienced athletes, within this age frame, are close to reaching their individual physical possibilities and may be less susceptible to further performance enhancement following caffeine ingestion [35].

In addition to its strengths, the present study has several limitations that should be addressed: (1) due to using commercially available products used in the study, we provided absolute doses of caffeine instead of the use of doses individualized to body mass. We have analyzed the results of this investigation taking into account the exact relative dose provided to each individual, which varied between 2.37 and 3.06 mg/kg for C + P and between 4.74 and 6.01 mg/kg for C + C, and we concluded that this small differences in relative doses did not affect the results of the investigation; (2) we did not evaluate blood caffeine concentration, thus we are unable to verify the level of blood caffeine concentration obtained with the use of chewing gum. However, previous investigations using similar caffeinated chewing gum and dosing of caffeine induced blood caffeine concentrations similar to those of caffeine capsules [9], and ergogenic effects of caffeine in sports are evident when using caffeinated chewing gum [10–17]; (3) the study analyzed the effects of caffeine intake on judo performance by only using two repetitions of the SJFT. Although this is the most common testing of judo performance in the literature [28], the use of other judo-specific testing such as the *judogi* grip strength test [23] or the number and duration of offensive actions during combat [24] may help to understand other potential ergogenic effects of caffeine in judo. Additionally, a higher number of the SJFTs, to simulate a more fatiguing competitive scenario may have helped to clarify the effects of acute caffeine intake on judo performance. Thus, more research is needed to determine an effective caffeine supplementation strategy using other performance tests, and taking into account various daily levels of caffeine consumption and training status. Additionally, future studies should explore the effectiveness of different doses of caffeine, provided from caffeinated gum and capsules.

## Conclusions

The results of the current investigation showed that ~ 2.7 mg/kg of caffeine (C + P) and ~ 5.4 mg/kg of caffeine (C + C) ingested via caffeinated chewing gum before two repetitions of the SJFT were ineffective to enhance the number of throws performed in this judo-specific test. In addition, these protocols of caffeine administration were also ineffective in inducing changes in the SJFT index, the rate of perceived exertion, heart rate and blood lactate concentration in elite judoists. From a practical perspective, the outcomes of this study and their comparison to previous literature suggests that, in elite judoists habituated to caffeine, doses lower than 6 mg/kg may be ineffective to improve judo-specific performance. One option to avoid the use of high doses of caffeine in judoists is to produce dishabituation to caffeine by reducing the amount of daily caffeine intake. Although the time course of re-sensitization to caffeine’s ergogenic effect after ceasing caffeine use is potentially impacted by the duration and extent of prior caffeine exposure [39], habitual users should cease caffeine ingestion at least 4 days prior to competition for dishabituation to occur [40, 41].

## Data Availability

The datasets used and/or analyzed during the current study are available from the corresponding author on reasonable request.
